# Description of a New Invented Spring Truss, to Guard against the Inconvenience of Ruptures

**Published:** 1815-02

**Authors:** J. Whitford

**Affiliations:** Surgical Instrument Maker and Cutler to St. Bartholomew's Hospital, &c.


*For the Medical and Physical Journal.*

### *Description of a new Invented Spring Truss, to guard against the Inconvenience of Ruptures;* by J. WHITFORD, Surgical Instrument Maker and Cutler to St. Bartholomew's Hospital, &c.

A VARIETY of Trusses has of late years been offered to the notice of the public, none differing, in my opinion, in the mode of applying the pad to the diseased part. When the pad of the truss is applied in the usual way, if the patient patient uses violent exertion, as jumping, coughing, sneezing, lifting heavy weights, &c. the part will, if the rupture be of long standing and very large, frequently slip from under, or before, the front part of the pad, which causes great uneasiness to the wearer, who, at the time of the accident, may not be at a convenient place to remove the truss, so as to return the prolapsed parts into the cavity of the abdomen. When this happens, it is generally attended with the most dangerous consequences by the pressure of the truss on the intestines, &c. I have, by long perseverance and practice in that part of the mechanism in trusses, endeavoured to remedy that defect, by preventing such unpleasant occurrences happening in future. It is not my intention to enter upon anatomical observations as to the structure of the human frame, but to confine myself principally to the mechanism of the truss, and to state as plainly as possible, to those who are afflicted with hernia, where the pad of the truss ought to be applied, in order to prevent the possibility of the ruptured part descending. The pads of trusses are in general applied considerably below where they ought to be, that is, too near to the os pubis. Hernia usually makes its first appearance nearly midway between the symphysis pubis and the anterior superior spine of the ilium; from thence it descends through the external opening which is situated just above the pubis, that is, having its basis resting upon the pubis, and its apex situated obliquely upwards and outwards; this is the part where the pad of the truss is usually applied; but it will appear evident on reflection, that, in order to effect a cure, it should be applied upon the upper opening, where the hernia first makes its appearance. It not unfrequently happens that the hernial sac, omentum, &c. has remained so long in the scrotum, that, by adhesion, or some other cause, it is impossible to return them into the cavity of the abdomen; it is necessary, in these cases, to inclose the parts in a strong bag truss, which will generally give ease by supporting the enormous weight that frequently occurs. The action and mode of applying the new truss to the ruptured part, will be seen by the Engravings introduced.

I shall now endeavour to point out the improvement that has been made in the truss.

Fig. 1 represents the spring uncovered, with a slight curve towards the neck of the spring from A to B, so as to allow the spring to adapt itself to the hollow of the groin from A to C. The neck of the spring is turned inwards and outwards, to act as a pair of spring forceps: the intention of the neck of the spring being bent, as exhibited, is, that the pressure shall be on the diseased part principally, and not to depend entirely on the power of the main spring from the back to the front, as is the case where great pressure is wanted to keep up the ruptured part. By this alteration you have a double spring, and on the inner spring is placed the pad, which pad is to be placed at the front part of the hernial ring; and, by the power of this spring, it obviates the necessity of having the circular spring so strong, the painful effects of which have been so much complained of. The great advantage this truss has over others is, that the pad is placed upwards instead of downwards; and, by this means, it is impossible for the hernia to pass by.

Fig. 2 shows the spring covered as it is to be worn: the pad is raised to a considerable thickness in the middle, and of very soft materials; the outer edge of the pad is left flat, as is represented at B, B, B. The rupture having been properly reduced, the projecting part of the pad is now to be placed exactly over the opening where the hernia descends, so that the pressure obtained by the extra spring is sufficient to press the parts so close together as to leave no doubt that, in the course of time, and more particularly in young subjects, an adhesion will take place, and in that case a cure will be obtained.

This fig. represents also where the pad is fixed to the inner spring, for in trusses in general the pads are fixed at the extreme neck of the spring; whereas this pad is now placed full three inches from the neck of the spring upwards, and, when properly applied, it is impossible for the ruptured part to descend.

It is likewise necessary to observe, that, in order to assist the truss in performing a cure, the rupture should never be suffered to descend, for it is by constant pressure a cure is likely to be obtained; to assist which I have made a bandage, which is called the night truss, without springs, and may be worn without any inconvenience.

Fig. 3 represents the truss applied on the right side, and secured by means of a front and under strap; the under strap is necessary in large and long-standing ruptures, as it will keep the truss more secure; but, in slight and recent hernia, it may be dispensed with, as the application of the pad in the new method will keep it steady.

*Directions for Applying the New-Invented Truss.*—When the intestine is returnable, the patient should lay himself on his back, bending his thigh a little upwards to relax the integuments; then the tumour is to be gently pressed upwards with his hand, until the hernia has ascended into the cavity of the abdomen; when this is done, apply the projecting part of the bolster of the spring pad to the opening where the the hernia descends; and, by keeping the parts pressed close together in this manner, a perfect cure will be obtained, especially if the disorder is recent, and in a child; and even in adults the ruptured part will become so contracted as not to admit of a falling-down of the intestine. Should they not be perfectly closed, if this method of applying the new truss is practised, I make no doubt it will never fail of success. J. WHITFORD.

*47, West Smithfield.*

